# Identifying viable *Neisseria gonorrhoeae* through validation and application of viability RT-PCR

**DOI:** 10.1128/spectrum.02605-25

**Published:** 2026-06-10

**Authors:** Sem E. H. Vellema, Christian J. P. A. Hoebe, Mayk Lucchesi, Paul H. M. Savelkoul, Petra F. G. Wolffs

**Affiliations:** 1 Department of Medical Microbiology, Infectious Diseases and Infection Prevention, Maastricht University Medical Centre+ (MUMC+) 199236, Maastricht, the Netherlands; 2 Care and Public Health Research Institute (CAPHRI), Maastricht University 5211, Maastricht, the Netherlands; 3 Department of Sexual Health, Infectious Diseases, and Environmental Health, Living Lab Public Health Mosa, South Limburg Public Health Service, Heerlen, the Netherlands; 4 Department of Social Medicine, Maastricht University, Faculty of Health, Medicine, and Life Sciences 82246, Maastricht, the Netherlands; ARUP Laboratories, Salt Lake City, Utah, USA

**Keywords:** *Neisseria gonorrhoeae*, cell viability, molecular methods, sexually transmitted diseases, viability PCR, bacterial culture

## Abstract

Gonorrhea, caused by *Neisseria gonorrhoeae* (NG), is rising globally alongside increasing antimicrobial resistance, including to first-line treatment. Standard diagnostics, such as nucleic acid amplification tests, cannot distinguish viable from non-viable bacteria, while culture—an inherent viability assessment—is challenged by the organism’s fastidious nature and environmental sensitivity. Molecular viability assays have not been applied to NG. This study aims to validate and apply a viability PCR (V-PCR) method for NG and perform an exploratory application on clinical samples to assess concordance with culture. Viability was assessed using PMAxx, a membrane-impermeable dye that binds nucleic acids in non-viable cells, enabling discrimination from viable cells. Technical validation used mixtures of viable and heat-killed NG with paired PMAxx-treated/PMAxx-untreated aliquots and subsequent *porA* qPCR to derive ΔCt values. In an exploratory application, clinical samples (anorectal, oropharyngeal, and urogenital) were tested using V-PCR and compared with culture. V-PCR eliminated 99.998% of DNA from non-viable NG, with a strong correlation (*R*²=0.96) across dilution series. Culture-positive clinical samples had a higher median viable load (3.92 log_10_ copies/mL, interquartile range [IQR]: 1.71–5.23) than culture-negative samples (0.87 log_10_ copies/mL, IQR: 0.38–2.69, *P* = 0.0003). Median viability percentage was significantly higher in culture-positive samples (16.92%, IQR: 0.41%–42.45%) versus culture-negative samples (1.23%, IQR: 0.00%–9.38%, *P* = 0.01). Overall, V-PCR reliably discriminated viable from non-viable NG in controlled experiments. In an exploratory application on clinical specimens, higher viable loads and viability percentages were associated with culture positivity. These findings support further evaluation of V-PCR as a tool to study factors influencing culture recovery; formal validation would be required before clinical implementation.

**IMPORTANCE:**

Gonorrhea affects an estimated 82 million people annually, with rising antimicrobial resistance threatening its treatability. Accurate diagnostics are essential, yet a diagnostic gap persists: standard PCR cannot distinguish viable from non-viable *Neisseria gonorrhoeae*, while culture—an inherent viability assessment—is challenged by the organism’s fastidious nature and environmental sensitivity. This limits accurate viability assessment, which is relevant to diagnostic practice, antimicrobial resistance surveillance, and broader research into gonococcal infection.

PMAxx-based viability PCR (V-PCR) has shown promise in other pathogens, including *Chlamydia trachomatis* and SARS-CoV-2, but had not been applied to *N. gonorrhoeae*. Here, V-PCR is validated for *N. gonorrhoeae* for the first time, demonstrating strong discrimination between viable and non-viable organisms with consistent performance across predefined viability ratios, and in an exploratory clinical application, viable load and viability percentage were significantly higher in culture-positive specimens. These findings establish a foundation for more accurate viability assessment in gonococcal diagnostics and research.

## INTRODUCTION

The sexually transmitted infection gonorrhea, caused by the bacterial pathogen *Neisseria gonorrhoeae* (NG), poses an increasing challenge and threat to public health. In 2020, an estimated 82 million cases occurred globally (1). In the EU, confirmed cases have steadily increased over recent years, reaching a record number of 70,881 confirmed cases in 2022. This increase of 59% compared to 2018 is likely an underestimation of the true situation (2). In recent years, the emergence of *N. gonorrhoeae* strains resistant to ceftriaxone, the global first-line antibiotic treatment, has raised significant concerns as well (3). This increasing antimicrobial resistance threatens to render gonococcal infections untreatable, potentially leading to serious complications such as pelvic inflammatory disease, ectopic pregnancy, infertility, or disseminated gonococcal disease.

In diagnosing gonorrhea, nucleic acid amplification tests (NAATs) have become the tool of choice due to their high sensitivity and specificity (4). While NAATs are excellent for detecting the presence of *N. gonorrhoeae*, they do not differentiate between viable and non-viable bacteria. Viability, in this context, refers to the organism’s ability to produce progeny, which is vital for successful culture.

While not primarily intended as a viability assay, successful bacterial culture as used in standard diagnostics inherently demonstrates the presence of viable organisms. Furthermore, culture remains crucial for antimicrobial susceptibility testing and surveillance of *N. gonorrhoeae* (5). However, *N. gonorrhoeae* is a highly fastidious microorganism that requires specific growth conditions and nutrients, making it sensitive to environmental changes (6). Consequently, the recovery rate of *N. gonorrhoeae* cultures is relatively low, presenting challenges for accurate viability assessment and antimicrobial resistance monitoring (7).

Molecular methods for assessing bacterial viability have become available as well (8–10). One method that can be easily integrated into nucleic acid-based testing is the use of viability PCR (V-PCR). This method involves the use of propidium monoazide (PMAxx) dye, which is a photoreactive, cell membrane-impermeable DNA-intercalating dye that is largely excluded from membrane-intact cells but can penetrate cells with compromised membranes. Upon light activation, it covalently cross-links to DNA, preventing subsequent PCR amplification. In contrast, DNA in membrane-intact bacterial cells remains unaffected, allowing for the selective detection of DNA from membrane-intact cells by PCR (11). Because PMAxx discrimination is based on membrane permeability, V-PCR provides an operational estimate of viability that reflects membrane integrity. Previous research has demonstrated its successful application in determining the viability of other pathogens, such as *Chlamydia trachomatis* and SARS-CoV-2, in clinical samples (12, 13). To date, no research has been conducted on the viability of *N. gonorrhoeae* in clinical samples using a molecular viability assay.

Therefore, this method validation study aims (i) to develop and validate a method for assessing the viability of *N. gonorrhoeae* and (ii) to perform an exploratory application of V-PCR on clinical specimens and to assess concordance between V-PCR results and culture.

## MATERIALS AND METHODS

### Technical validation

**Fig 1:**
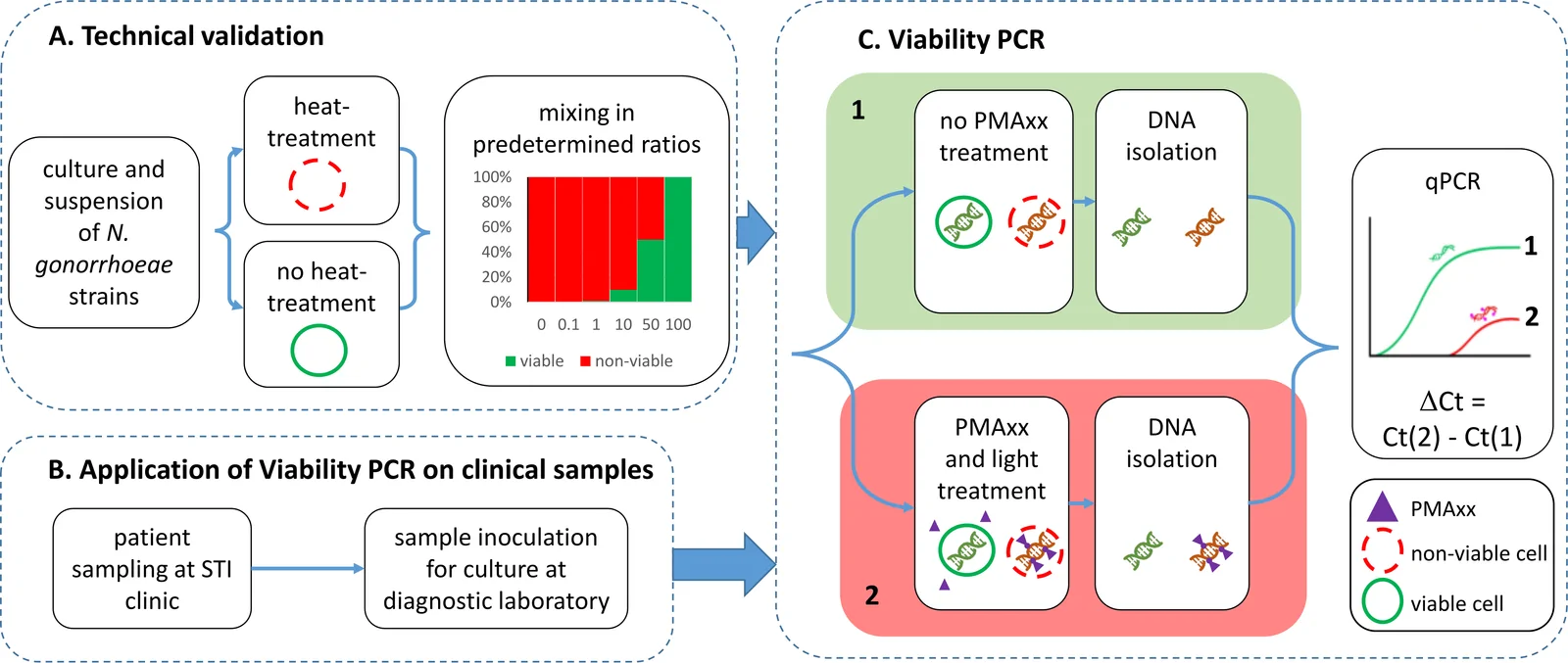
Schematic representation of sample processing for (**A**) the dilution series of cultured *N. gonorrhoeae* strains for technical validation and (**B**) clinical samples collected for diagnostic purposes at STI clinics. (**C**) Representation of the viability assay used for both panels **A** and **B**, where aliquots of PMAxx-treated and PMAxx-untreated materials subsequently undergo PCR.

For the technical method validation of V-PCR, a dilution series of predefined viability ratios of *N. gonorrhoeae* strains was produced (Fig. 1A). *N. gonorrhoeae* ATCC reference strains 49226 and 19424 (ATCC, Manassas, VA), and three clinical strains isolated from clinical urethral specimens (Table S1) were streaked on Chocolate II Agar with IsoVitaleX supplement (BD, Sparks, MD) and incubated as three independent cultures per strain (24 h, 37°C, 5% CO_2_). After culture, bacterial colonies were suspended in a saline solution. In order to achieve comparable cell densities, suspensions were adjusted to an optical density (OD_600_) of 1.5 and then divided into two aliquots. One aliquot was heat-inactivated at 95°C for 10 min at 300 rpm to produce exclusively non-viable material using an Eppendorf Thermo Mixer (Eppendorf, Hamburg, Germany). Aliquots were then mixed in a series of predetermined ratios of heat-treated and untreated material, with untreated (=viable) material representing 0%, 0.1%, 1%, 10%, 50%, and 100% of each mixture, respectively. Each mixture was then split into two aliquots: one aliquot was treated with PMAxx dye and one aliquot was left untreated, after which both aliquots underwent qPCR for the *porA* gene. Heat-treated aliquots were inoculated on Chocolate II Agar with IsoVitaleX supplement culture plates (BD) to confirm inactivation of bacterial strains.

### Viability assay and DNA extraction

For the viability assay, culture samples were treated with PMAxx dye (Biotium, Fremont, CA) (Fig. 1C). This photoreactive dye was used to distinguish between viable and non-viable bacteria. PMAxx is membrane impermeable and selectively enters cells with compromised membranes, which are typically considered non-viable. Once inside these cells, PMAxx intercalates with its DNA content. A volume of PMAxx (20 mM) appropriate for a final concentration of 50 μM (in accordance with the manufacturer’s recommendations) was added to 400 μL of bacterial suspension (technical validation) or eSwab medium (clinical sample). PMAxx-treated and PMAxx-untreated aliquots were prepared for each to allow for comparison. eSwabs were vortexed at low speed before aliquoting.

PMAxx-treated aliquots were incubated at room temperature in the dark for 10 min, allowing the dye to penetrate non-viable cells and bind to available DNA. Subsequently, samples were exposed for 10 min to blue LED light (470 nm) using a PMA-Lite LED Photolysis Device (Biotium) to achieve photoactivation. This light exposure causes intercalated PMAxx to form covalent bonds with DNA, effectively modifying it and inhibiting its amplification in subsequent PCR reactions.

Genomic DNA was then extracted from both PMAxx-treated and PMAxx-untreated aliquots using the MagNa Pure 96 system (Roche Diagnostics, Rotkreuz, Switzerland) for automated purification of nucleic acids. Extraction was performed using the MagNA Pure 96 DNA and Viral NA Small Volume Kit (Roche Diagnostics) using the Pathogen Universal 200 Protocol (Roche Diagnostics). Samples were eluted in 50 μL elution buffer and diluted with 50 μL water for molecular biology (VWR International, Radnor, PA).

### PCR amplification

Quantitative PCR targeting the *N. gonorrhoeae*-specific *porA* pseudogene was performed on both PMAxx-treated and PMAxx-untreated aliquots (Fig. 1C). Each PCR reaction was prepared with a total volume of 25 µL, consisting of 12.5 µL of TaqPath qPCR Master Mix (Thermo Fisher Scientific, Waltham, MA), 10 µL of purified target DNA, and 2.5 µL primer/probe mix. The primer/probe mix stock consisted of 40 µL of 100 μM forward primer (5′-CAGCATTCAATTTGTTCCGAGTC-3′), 40 µL of 100 μM reverse primer (5′-ATCACTCGCTCTGCCGAG-3′), 5 µL of 100 μM probe (6FAM-5′-CGCCTATACGCCTGCTACTTTCACGC-3′-BHQ1), and 390 µL of nuclease-free water. Amplification and detection were conducted with the ABI PRISM 7900 Sequence Detection System (Applied Biosystems, Waltham, MA). The PCR amplification program consisted of an initial annealing step at 95°C for 15 min, followed by 42 cycles of denaturation at 95°C for 15 sec and extension at 60°C for 1 min, yielding a 244 bp amplicon.

### Analytical limit of detection

An analytical limit of detection (LoD) assessment was performed for our *porA* qPCR assay using a 10-fold dilution series (10^−1^ to 10^−8^) of DNA eluate prepared from a *Neisseria gonorrhoeae* ATCC 49226 bacterial suspension standardized to OD_600_ = 0.5. Each dilution was tested across nine independent dilution curves. Detection probability at each dilution was recorded, and the LoD95 was defined as the highest dilution at which ≥95% of replicates were detected. To approximate biological input at the LoD95, 50 µL of the corresponding bacterial dilution was plated on chocolate agar to obtain CFU counts, which were converted to an approximate CFU equivalents per PCR reaction based on the extraction and elution volumes (provided in Table S1 and Fig S1).

### Clinical sample collection and selection

Clinical samples were previously collected from patients visiting two STI clinics of the Public Health Services (GGD) in the province of Limburg, the Netherlands. Patients were initially tested for *N. gonorrhoeae* using the Cobas 6800 CT/NG assay (Roche Diagnostics). Those testing positive had additional swabs taken for culture from the anatomical site in question and were included for viability testing (Fig. 1B). Culture samples included urethral, vaginal, anorectal, and oropharyngeal specimens. After collection, swabs were stored in eSwab liquid transport medium (Copan Diagnostics, Murrieta, CA) and stored at 4°C prior to and during transport to the diagnostic laboratory. Samples were collected and processed over a 5-month period in 2024 (*n* = 147). Samples were then excluded if residual material was insufficient or if the time interval between laboratory receipt and PMAxx processing exceeded 12 h (excluded *n* = 60), leaving 87 samples for further analysis.

### Clinical diagnostics

After collection, samples were inoculated at the clinical diagnostics laboratory onto chocolate agar plates supplemented with Vitox (Oxoid, Basingstoke, United Kingdom) and incubated at 37°C in a 5% CO_2_ atmosphere. The plates were monitored for colony growth over a period of 5 days. In the event of colony growth, species identification was carried out using the VITEK MS system (BioMerieux, Marcy-l’Étoile, France), which utilizes matrix-assisted laser desorption/ionization time-of-flight mass spectrometry for identification of *N. gonorrhoeae*.

### Data analysis

Cycle threshold (Ct) values from the qPCR were compared between PMAxx-treated and PMAxx-untreated aliquots. Statistical analysis comparing viability between culture-positive and culture-negative samples was conducted using the Wilcoxon rank-sum test, given the non-normal distribution of the data.

Clinical samples for which more than 12 h had elapsed between laboratory receipt and PMAxx processing were excluded from analysis. Resultant sample and patient characteristics are shown in Table 1. This exclusion criterion was implemented to minimize the impact of time as a confounding factor in the viability assessment.

**TABLE 1:** Summary of participant and sample characteristics (*n* = 87)

Characteristics		Value
Age (years), median (IQR)		33 (23.5–42.5)
Gender, *n* (%)		
Female		16 (18.4)
Male		70 (80.5)
Non-binary/other		1 (1.1)
Sample site, *n* (%)		
Anorectal		26 (29.9)
Oral		12 (13.8)
Urethral		37 (42.5)
Vaginal		12 (13.8)
Bacterial culture result, *n* (%)		
Negative		30 (34.5)
Positive		57 (65.5)

In samples where no Ct values were detected through PCR, the samples were imputed with a Ct value of 42, allowing for further analysis. Cases with Ct values imputed to 42 in both PMAxx-treated and PMAxx-untreated aliquots were treated as non-informative and assigned 0% viability. Where ΔCt-based calculations yielded viability estimates >100%, values were capped at 100%.

Sensitivity analyses were performed to assess the robustness of culture-positive versus culture-negative comparisons to the handling of imputed and capped values. Wilcoxon rank-sum comparisons of log_10_ viable load and viability percentage were repeated after (A) excluding samples in which both PMAxx-treated and PMAxx-untreated reactions were imputed (both Ct values at the imputation limit); (B) applying the same exclusion as (A) and additionally excluding observations with raw viability estimates >100% (i.e., those requiring capping at 100%); and (C) excluding any sample with imputation in either reaction (measured Ct pairs only). Full sensitivity analysis results are reported in Table S3.

For interpretability, we additionally performed an exploratory ROC-based dichotomization of V-PCR outputs against culture and report the resulting 2 × 2 tables and metrics in Table S4.

## RESULTS

### Technical validation of viability PCR

The first aim of this study was to validate the capacity of the V-PCR to distinguish between intact (viable) *N. gonorrhoeae* and nucleic acids still present in cell membrane-compromised non-viable *N. gonorrhoeae*.

**Fig 2:**
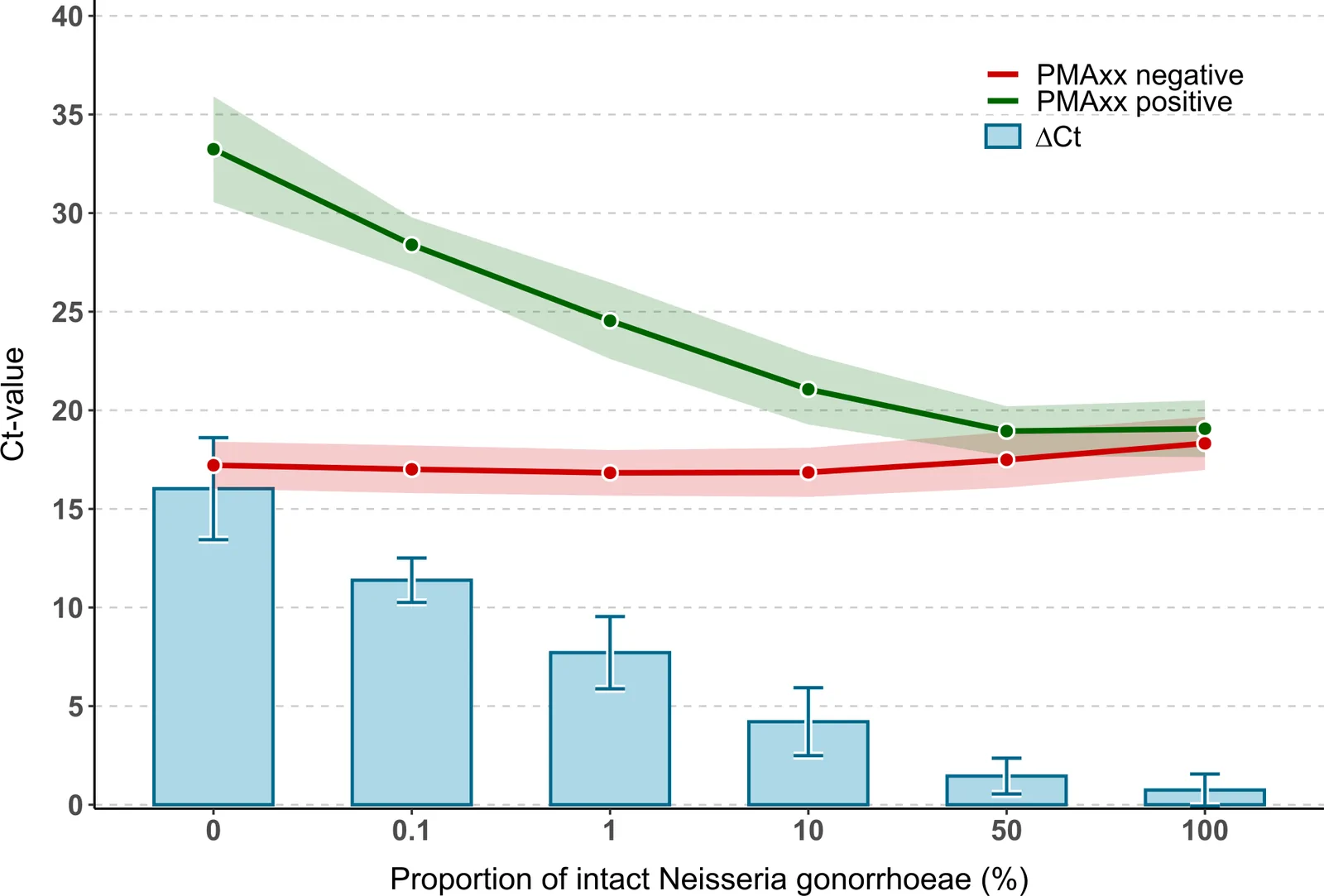
Validation results of the viability PCR in cultured clinical (*n* = 3) and reference (*n* = 2) *N. gonorrhoeae* strains. The *x*-axis shows a dilution series of predefined mixtures of intact and heat-killed *N. gonorrhoeae*. Each mixture was split into PMAxx-treated and PMAxx-untreated aliquots, shown by the green and red lines, respectively. Ct values are presented as mean ± SD across 15 independent biological replicates (five strains × three cultures). ΔCt values were calculated from the differences in Ct values between PMAxx-treated and PMAxx-untreated samples for each mixture.

To achieve this, V-PCR was performed on dilution series consisting of 0%, 0.1%, 1%, 10%, 50%, and 100% intact *N. gonorrhoeae* for both PMAxx- and non-PMAxx-treated samples in triplicate. Obtained Ct values were used to calculate ΔCt values per dilution (Fig. 2).

The V-PCR showed mean (±SD) ΔCt values of 16.03 ± 2.59 (0%), 11.38 ± 1.13 (0.1%), 7.71 ± 1.83 (1%), 4.21 ± 1.72 (10%), 1.45 ± 0.91 (50%), and 0.74 ± 0.81 (100%). A mean ΔCt of 16.03 corresponds to suppression of approximately 99.998% of amplifiable DNA in samples with no viable *N. gonorrhoeae*. Linear regression analysis performed on log-transformed ΔCt values of the dilution series resulted in an *R*^2^-value of 0.96. Strain-level validation results are shown in Fig. S2.

An analytical limit of detection assessment of the *por*A qPCR showed detection 9/9 replicates at the 10^−5^ eluate dilution and in 4/9 replicates at 10^−6^. Therefore, the LoD95 was defined as 10^−5^ under our assay conditions. Plating of the corresponding bacterial dilution yielded 46 CFU per 50 µL, corresponding to an LoD95 of approximately 15 CFU-equivalents per PCR reaction (see Table S2 and Fig S1).

### Absolute quantification: viable load

The second aim of the study was to perform an exploratory application of V-PCR on clinical specimens and assess concordance between V-PCR results and culture. The analysis included 87 samples that met the inclusion criteria (≤12 h between laboratory receipt and PMAxx processing and sufficient residual material for testing).

**Fig 3:**
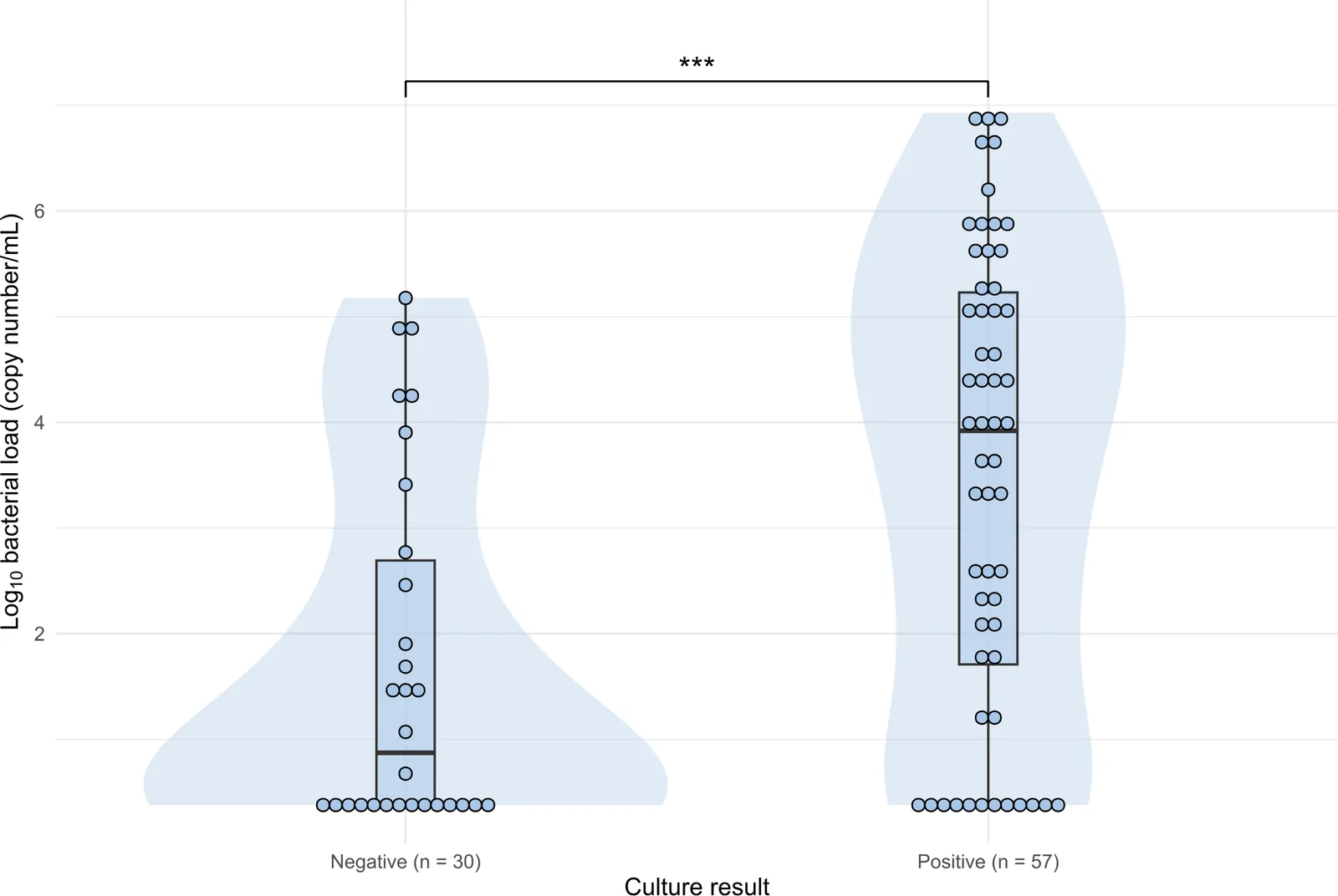
Viable bacterial load in clinical samples as determined by viability PCR, categorized by culture result. The scatterplot displays log_10_ copy numbers per milliliter for each sample, including boxplots representing the median and interquartile ranges, with violin plots showing data distribution. Asterisks (***) denote a statistically significant difference between groups (*P* value of <0.001).

Ct values of PMAxx-treated samples were converted to gene equivalent copy numbers per milliliter using data from a previously published PCR assay for the *porA* gene, assuming comparable qualities with our *porA* assay. Loads were log_10_-transformed to normalize the distribution and stabilize variance. Viable bacterial loads were evaluated by culture result, with 57 culture-positive samples and 30 culture-negative samples (Fig. 3). The median log_10_ viable load for culture-positive samples was 3.92 log_10_ copies/mL (interquartile range [IQR]: 1.71–5.23), whereas culture-negative samples exhibited a median log_10_ viable load of 0.87 log_10_ copies/mL (IQR: 0.38–2.69). A statistically significant difference was observed between the two groups (*P* value = 0.0003) using the Wilcoxon rank-sum test.

Sensitivity analyses excluding imputed and/or bounded observations yielded consistent results, with culture-positive samples remaining significantly higher in log_10_ viable load across all scenarios (see Table S3).

### Relative quantification: viability percentage

**Fig 4:**
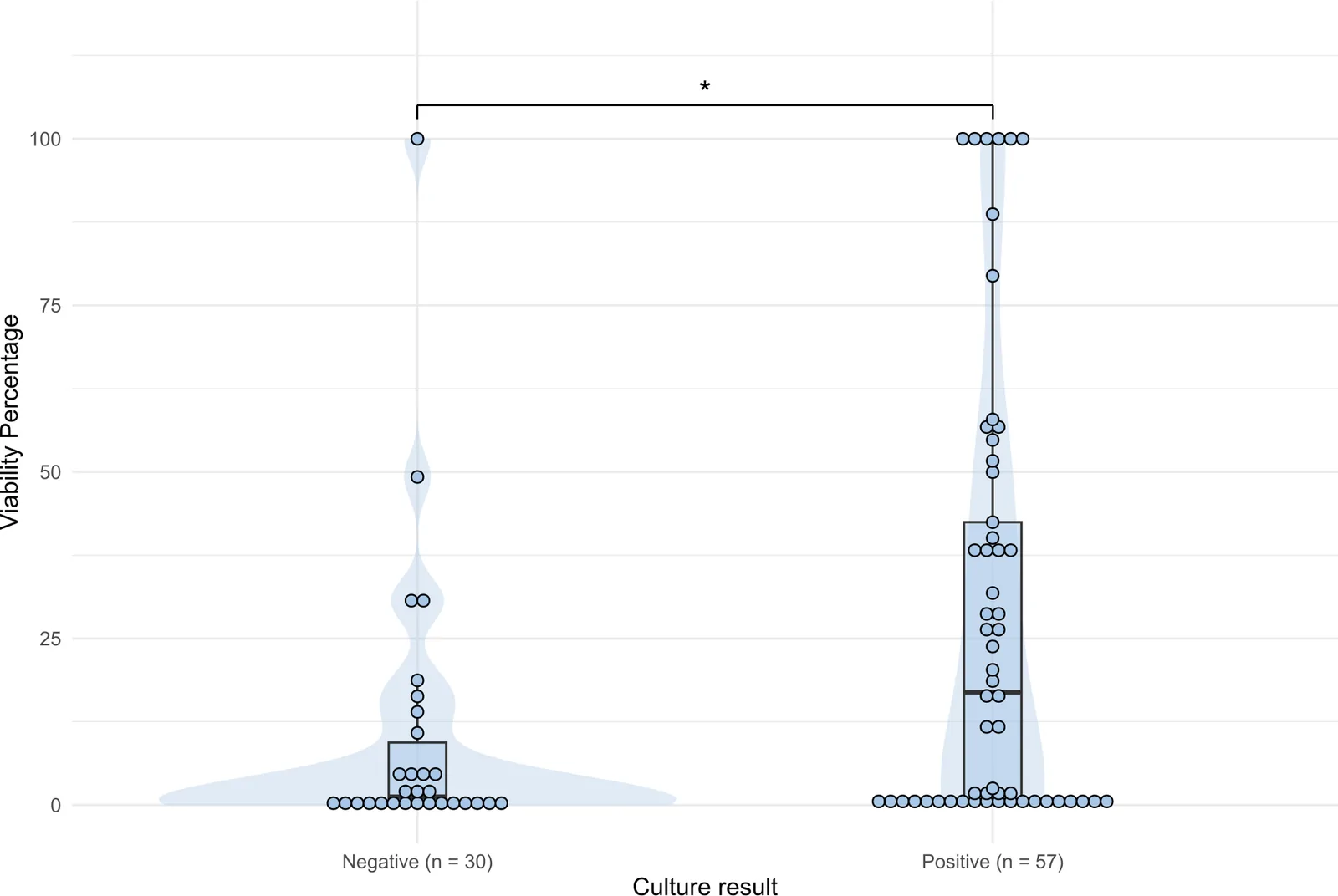
Viability percentages of clinical samples determined by viability PCR, categorized by culture result. The scatterplot displays individual viability percentages, with violin plots showing data distribution and boxplots indicating the median and interquartile ranges. The asterisk (*) denotes a statistically significant difference between groups (*P* value of 0.01).

Results of the viability PCR on clinical samples were also analyzed using a ΔCt-based relative quantification. For each sample, the PMAxx-untreated aliquot was used as the within-sample reference, and the corresponding PMAxx-treated aliquot was compared to it to estimate the proportion of amplifiable DNA remaining after PMAxx treatment. Viability percentage was calculated, and values exceeding 100% (which can occur due to minor technical variation yielding negative ΔCt values) were capped at 100%. This approach allowed for per-sample quantification of viable *N. gonorrhoeae* relative to the total *N. gonorrhoeae* detected by PCR. Viability percentages were then grouped by culture result (Fig. 4). The median viability percentage for culture-positive samples was 16.92% (IQR: 0.41–42.45), whereas for culture-negative samples, the median was 1.23% (IQR: 0.00%–9.38%). A comparison of these two categories using the Wilcoxon rank-sum test yielded a statistically significant *P* value of 0.01.

Sensitivity analyses excluding imputed and/or bounded observations yielded consistent results, with viability percentages remaining higher in culture-positive than culture-negative samples across all scenarios (see Table S3).

## DISCUSSION

Our study is the first to demonstrate the successful use of viability PCR to distinguish between viable and non-viable *N. gonorrhoeae*, with validation showing 99.998% elimination of non-viable DNA. Furthermore, the dilution series demonstrated an *R*^2^ value of 0.96, suggesting that Ct values generated by the V-PCR are highly consistent with the expected log-linear relationship across the dilution series.

This implies that V-PCR is effective at discriminating between viable and non-viable *N. gonorrhoeae* and is consistent among differing ratios of live and dead *N. gonorrhoeae*, meaning that it may be used to accurately determine unknown sample concentrations of viable *N. gonorrhoeae*.

In the exploratory application of V-PCR on clinical specimens, our results revealed significant differences in viable bacterial load between culture-positive and culture-negative samples. Samples in which bacterial culture was successful contained both a statistically significantly higher absolute viable bacterial load as well as a higher proportion of viable *Neisseria gonorrhoeae*. These findings were directionally consistent in sensitivity analyses that varied handling of imputed and/or bounded values. This is in line with expectations, as bacterial culture is fundamentally a viability measurement, relying on the ability of bacteria to reproduce under suitable conditions.

However, multiple cases where culture was negative exhibited moderate viable loads and viability percentages. This may reflect physiological states in which culturability is reduced despite preserved membrane integrity (e.g., viable-but-non-culturable states or recent antibiotic exposure [9]), as V-PCR operationalizes viability primarily via membrane integrity (14, 15). Furthermore, in a number of samples with positive culture results, we detected absent or low viable loads. This discordance may be driven by preanalytical factors, including specimen matrix effects and variability in post-receipt handling time prior to PMAxx treatment, which can shift reactions toward the assay’s detection limit and thereby yield non-informative viability estimates.

In some samples with positive culture results, viability percentages of 0 or near 0 were measured. Notably, many culture-positive samples with such viability percentages occurred when PCR signal was below the detection limit in both the PMAxx-treated and PMAxx-untreated aliquots, yielding a non-informative viability estimate rather than clear evidence of absent viability. A number of these samples, however, also showed correspondingly moderate to low viable loads as determined by V-PCR, so even though the viable percentage in these samples may be low, the absolute number of viable bacteria present may still be significant enough for successful culture (16). These observations underscore the complexity of *N. gonorrhoeae* culture and the potential limitations of culture-based diagnostics.

The ability to differentiate between viable and non-viable *N. gonorrhoeae* through V-PCR has significant implications in several areas. In clinical diagnostics routines, utilizing V-PCR as a preculture screening test on incoming samples in order to identify viable *N. gonorrhoeae* may improve culture recovery rates. Furthermore, identifying solely viable *N. gonorrhoeae* may nuance actual infection status at different anatomical sites and associated transmission risks, which may help inform research questions related to persistence and transmission potential.

In research settings, this information could also be useful in researching autoinoculation processes and thereby aiding in the development of more targeted prevention strategies (17). Application of V-PCR in longitudinal studies could provide insights into the viability of *N. gonorrhoeae* during infection and antibiotic treatment, which may prove useful in optimizing treatment regimens and understanding the development of antibiotic resistance.

A notable limitation was variability in the time from laboratory receipt to PMAxx treatment, as V-PCR was performed after culture inoculation. Delays during this post-receipt interval may affect measured viability and can shift low-load specimens toward the assay’s detection limit.. Future studies should aim to minimize this time gap to better correlate V-PCR results with culture outcomes. In contrast, in several cases where *N. gonorrhoeae* went (nearly) undetected by PCR, culture was still successful, indicating that there may still be some optimization possible for the PCR assay used. Discrepant results are expected mainly near the assay’s detection limit, so continued evaluation of analytical sensitivity in clinical matrices may help interpret borderline samples.

Further research is needed to evaluate V-PCR performance across diverse patient populations and anatomical sites. Future studies should assess the relationship between viable bacterial load and clinical outcomes at different anatomical sites, which could provide insights into the transmission dynamics of *N. gonorrhoeae* between anatomical sites and the subsequent prognostic value of V-PCR.

In conclusion, V-PCR shows promise as a valuable tool for *N. gonorrhoeae* diagnostics and research. By providing a more accurate picture of viable bacterial load versus non-viable bacterial load, this technique could aid in optimizing diagnostic routines, attenuating clinical decision-making, and furthering our understanding of gonococcal infections, contributing to more effective antimicrobial and diagnostic stewardship.

## Data Availability

The data underlying this study are openly available at https://doi.org/10.5281/zenodo.20397180.

## SUPPLEMENTAL MATERIAL

Tables S1 to S4; Figures S1 and S2 spectrum.02605-25-s0001.docx Table S1: Characteristics of cultured clinical strains. Table S2: LoD Ct-values generated through porA qPCR of 9 dilution series of DNA isolate from cultured Neisseria gonorrhoeae ATCC 49226. Table S3: Sensitivity analyses for group differences by culture results. Table S4: ROC-derived 2×2 tables for V-PCR outputs versus culture. Figure S1: Technical validation per Neisseria gonorrhoeae strain. Figure S2: Mean dilution curve of 9 independent dilution series of DNA isolate from cultured *Neisseria gonorrhoeae* ATCC 49226 for LoD.

ASM does not own the copyrights to Supplemental Material that may be linked to, or accessed through, an article. The authors have granted ASM a non-exclusive, world-wide license to publish the Supplemental Material files. Please contact the corresponding author directly for reuse.

## References

[1] World Health Organization. 2024. Implementing the global health sector strategies on HIV, viral hepatitis and sexually transmitted infections, 2022–2030: report on progress and gaps 2024. Geneva

[2] European Centre for Disease Prevention and Control. 2024. Gonorroea: Annual Epidemiological Report for 2022. Stockholm

[3] European Centre for Disease Prevention and Control. 2024. Gonococcal antimicrobial susceptibility surveillance in the European Union/European Economic Area, 2022. Stockholm

[4] Unemo M, Ross J, Serwin AB, Gomberg M, Cusini M, Jensen JS. 2020. 2020 European guideline for the diagnosis and treatment of gonorrhoea in adults. Int J STD AIDS 2020:956462420949126. doi:10.1177/0956462420949126 33121366

[5] Weston EJ, Wi T, Papp J. 2017. Strengthening global surveillance for antimicrobial drug-resistant neisseria gonorrhoeae through the enhanced gonococcal antimicrobial surveillance program. Emerg Infect Dis 23:S47–52. doi:10.3201/eid2313.170443 29155673 PMC5711314

[6] Green LR, Cole J, Parga EFD, Shaw JG. 2022. *Neisseria gonorrhoeae* physiology and pathogenesis. Adv Microb Physiol 80:35–83. doi:10.1016/bs.ampbs.2022.01.002 35489793

[7] Nash EE, Pham CD, Raphael B, Learner ER, Mauk K, Weiner J, Mettenbrink C, Thibault CS, Fukuda A, Dobre-Buonya O, Black JM, Johnson K, Sellers K, Schlanger K, SURRG Working Group. 2021. Impact of anatomic site, specimen collection timing, and patient symptom status on *Neisseria gonorrhoeae* culture recovery. Sex Transm Dis 48:S151–S156. doi:10.1097/OLQ.0000000000001540 34433797 PMC9125530

[8] Cangelosi GA, Meschke JS. 2014. Dead or alive: molecular assessment of microbial viability. Appl Environ Microbiol 80:5884–5891. doi:10.1128/AEM.01763-14 25038100 PMC4178667

[9] Trinh KTL, Lee NY. 2022. Recent methods for the viability assessment of bacterial pathogens: advances, challenges, and future perspectives. Pathogens 11:1057. doi:10.3390/pathogens11091057 36145489 PMC9500772

[10] Baymiev AnKh, Baymiev AlKh, Kuluev BR, Shvets KYu, Yamidanov RS, Matniyazov RT, Chemeris DA, Zubov VV, Alekseev YaI, Mavzyutov AR, Ivanenkov YaA, Chemeris AV. 2020. Modern approaches to differentiation of live and dead bacteria using selective amplification of nucleic acids. Microbiology (Reading, Engl) 89:13–27. doi:10.1134/S0026261720010038

[11] Fittipaldi M, Nocker A, Codony F. 2012. Progress in understanding preferential detection of live cells using viability dyes in combination with DNA amplification. J Microbiol Methods 91:276–289. doi:10.1016/j.mimet.2012.08.007 22940102

[12] Janssen KJH, Hoebe CJPA, Dukers-Muijrers NHTM, Eppings L, Lucchesi M, Wolffs PFG. 2016. Viability-PCR shows That NAAT detects a high proportion of DNA from non-viable *Chlamydia trachomatis*. PLoS One 11:e0165920. doi:10.1371/journal.pone.0165920 27812208 PMC5094775

[13] Veugen JMJ, Schoenmakers T, van Loo IHM, Haagmans BL, Leers MPG, Lamers MM, Lucchesi M, van Bussel BCT, van Mook WNKA, Nuijts RMMA, Savelkoul PHM, Dickman MM, Wolffs PFG. 2024. Advancing COVID-19 diagnostics: rapid detection of intact SARS-CoV-2 using viability RT-PCR assay. Microbiol Spectr 12:e0016024. doi:10.1128/spectrum.00160-24 39037224 PMC11370235

[14] Liu J, Yang C, Cheng C, Zhang C, Zhao J, Fu C. 2021. *In vitro* antimicrobial effect and mechanism of action of plasma-activated liquid on planktonic *Neisseria gonorrhoeae*. Bioengineered 12:4605–4619. doi:10.1080/21655979.2021.1955548 34320914 PMC8806901

[15] Oliver JD. 2010. Recent findings on the viable but nonculturable state in pathogenic bacteria. FEMS Microbiol Rev 34:415–425. doi:10.1111/j.1574-6976.2009.00200.x 20059548

[16] Chow EPF, Tabrizi SN, Phillips S, Lee D, Bradshaw CS, Chen MY, Fairley CK. 2016. *Neisseria gonorrhoeae* bacterial DNA load in the pharynges and saliva of men who have sex with men. J Clin Microbiol 54:2485–2490. doi:10.1128/JCM.01186-16 27413195 PMC5035428

[17] Dukers-Muijrers NHTM, Wolffs PFG, Eppings L, Götz HM, Bruisten SM, Schim van der Loeff MF, Janssen K, Lucchesi M, Heijman T, van Benthem BH, van Bergen JE, Morre SA, Herbergs J, Kok G, Steenbakkers M, Hogewoning AA, de Vries HJ, Hoebe CJPA. 2016. Design of the FemCure study: prospective multicentre study on the transmission of genital and extra-genital *Chlamydia trachomatis* infections in women receiving routine care. BMC Infect Dis 16:381. doi:10.1186/s12879-016-1721-x 27502928 PMC4977887

