# Supplementary material for: Identifying viable Neisseria gonorrhoeae through validation and application of viability RT-PCR
Source: Microbiol Spectr. 2026 Jun 10;14(7):e02605-25. doi: 10.1128/spectrum.02605-25 (PMC13340066; doi:10.1128/spectrum.02605-25)
Supplement: Tables S1 to S4; Figures S1 and S2 — Table S1: Characteristics of cultured clinical strains. Table S2: LoD Ct-values generated through porA qPCR of 9 dilution series of DNA isolate from cultured Neisseria gonorrhoeae ATCC 49226. Table S3: Sensitivity analyses for group differences by culture results. Table S4: ROC-derived 2×2 tables for V-PCR outputs versus culture. Figure S1: Technical validation per Neisseria gonorrhoeae strain. Figure S2: Mean dilution curve of 9 independent dilution series of DNA isolate from cultured Neisseria gonorrhoeae ATCC 49226 for LoD. [file spectrum.02605-25-s0001.docx]

**SUPPLEMENTAL MATERIAL**

**Table S1. Characteristics of cultured clinical strains**

**Table S2. LoD Ct-values generated through porA qPCR of 9 dilution series of DNA isolate from cultured Neisseria gonorrhoeae ATCC 49226**

**Table S3. Sensitivity analyses for group differences by culture results**

**Figure S1. Technical validation per *Neisseria gonorrhoeae* strain**

**Figure S2. Mean dilution curve of 9 independent dilution series of DNA isolate from cultured *Neisseria gonorrhoeae* ATCC 49226 for LoD**

**Table S4. ROC-derived 2×2 tables for V-PCR outputs versus culture**

**Table S1. Characteristics of cultured clinical strains**

|  | **Date of collection (month-year)** | **Patient gender** | **Sample type** | **Azithromycin MIC** | **Ceftriaxon MIC** |
| --- | --- | --- | --- | --- | --- |
| **Clinical strain 1** | 09-2021 | M | Urethral swab | 2 | 0.004 |
| **Clinical strain 2** | 04-2016 | M | Urethral swab | 0.094 | 0.004 |
| **Clinical strain 3** | 01-2021 | M | Urethral swab | 0.064 | 0.003 |

**Table S2. LoD Ct-values generated through *porA* qPCR of 9 dilution series of DNA isolate from cultured *Neisseria gonorrhoeae* ATCC 49226**

| Dilution step | Dilution series 1 | Dilution series 2 | Dilution series 3 | Dilution series 4 | Dilution series 5 | Dilution series 6 | Dilution series 7 | Dilution series 8 | Dilution series 9 | Dilution series mean |
| --- | --- | --- | --- | --- | --- | --- | --- | --- | --- | --- |
| 10^-1 | 22,35 | 22,30 | 22,34 | 22,34 | 22,23 | 22,37 | 22,17 | 22,16 | 22,12 | 22,26 |
| 10^-2 | 26,02 | 26,26 | 26,04 | 25,97 | 25,91 | 25,92 | 25,78 | 25,91 | 25,79 | 25,95 |
| 10^-3 | 29,12 | 29,38 | 29,39 | 29,13 | 29,36 | 29,15 | 29,11 | 29,12 | 29,18 | 29,22 |
| 10^-4 | 33,25 | 33,01 | 32,99 | 32,85 | 33,08 | 32,35 | 33,20 | 32,85 | 32,71 | 32,92 |
| 10^-5 | 35,53 | 36,31 | 36,41 | 36,52 | 35,84 | 35,83 | 36,87 | 35,99 | 37,78 | 36,34 |
| 10^-6 | N.D. | 39,83 | N.D. | N.D. | 37,56 | N.D. | N.D. | 39,52 | 40,15 | 38,97 |
| 10^-7 | N.D. | N.D. | N.D. | N.D. | N.D. | N.D. | N.D. | N.D. | N.D. | N.D. |
| 10^-8 | N.D. | N.D. | N.D. | N.D. | N.D. | N.D. | N.D. | N.D. | N.D. | N.D. |

**Figure S1. Mean dilution curve of 9 independent dilution series of DNA isolate from cultured *Neisseria gonorrhoeae* ATCC 49226 for LoD**

A 10-fold dilution series of DNA eluate (10^-1^-10^-8^)was prepared from DNA extracted from 200 µL bacterial suspension of *Neisseria gonorrhoeae* ATCC strain 49226 calibrated to OD_600_ = 0.5. Across nine independent dilution curves (slope -3.38, intercept 19.11, R² = 0.998) detection was 9/9 for dilution 10^-5^ and 4/9 for dilution 10^-6^. Therefore, the LoD95 was defined as 10^-5^ under our assay conditions.

To approximate biological input, plating 50 µL of the 10^-5^ bacterial dilution yielded 46 CFU, corresponding to ±15 CFU-equivalents per reaction at the 10^-5^ eluate dilution (based on our extraction/elution volumes).

**Table S3. Sensitivity analyses for group differences by culture result.** Wilcoxon rank-sum tests comparing culture-negative vs culture-positive samples.

| **Dataset** | **Definition** | **N total** | **N culture negative** | **N culture positive** | **log_10_ viable load per mL (W)** | **log_10_ viable load per mL (p-value)** | **Viability % (W)** | **Viability % (p-value)** |
| --- | --- | --- | --- | --- | --- | --- | --- | --- |
| Primary | Main analytic dataset | 87 | 30 | 57 | 460 | 0.00036 | 569 | 0.010 |
| A | Excludes samples in which both reactions were imputed (both Ct values at the imputation limit) | 68 | 21 | 47 | 221 | 0.00030 | 314 | 0.017 |
| B | Excludes samples in which both reactions were imputed and excludes observations with raw viability >100% | 61 | 20 | 41 | 185 | 0.00056 | 270 | 0.032 |
| C | Excludes any imputed Ct in PMA+ or PMA− | 60 | 15 | 45 | 158 | 0.00225 | 193 | 0.014 |

Sensitivity analyses assessed the robustness of culture-positive versus culture-negative comparisons to two data-handling steps used for ΔCt-derived viability estimates: imputation of non-detect Ct values to the assay limit and capping of viability percentage values >100% at 100%.

Wilcoxon rank-sum comparisons of log_10_ viable load and viability percentage were repeated after: (A) excluding samples in which both PMAxx-treated and untreated reactions were imputed (both Ct values at the imputation limit), (B) applying the same exclusion as (A) and additionally excluding observations with raw viability estimates >100% (i.e., those requiring capping at 100%), and (C) excluding any sample with imputation in either reaction (measured Ct pairs only).

Across these three sensitivity analyses that excluded imputed and/or capped observations, culture-positive samples consistently showed statistically significant higher viable loads and higher viability percentages than culture-negative samples, indicating that the main findings are robust and not driven by boundary handling or Ct imputation.

**
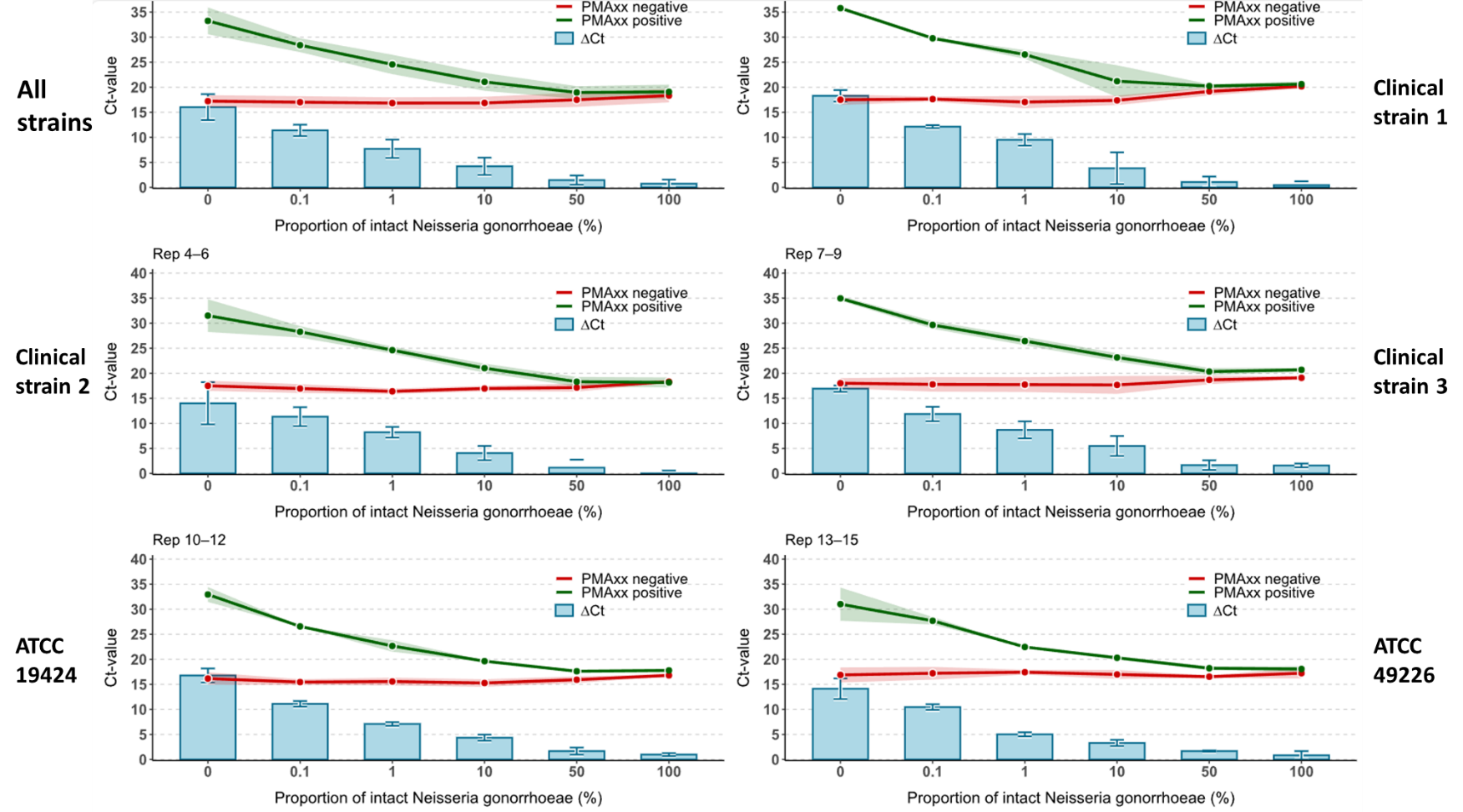
**

**Figure S2 Technical validation per *Neisseria gonorrhoeae* strain.** The x-axis shows a dilution series of predefined mixtures of intact and heat-killed *N. gonorrhoeae*. Each mixture was split into PMAxx-treated and untreated aliquots, shown by the green and red lines, respectively. Ct-values are presented as mean ± SD across 3 independent biological replicates for each strain. ΔCt-values were calculated from the differences in Ct-value between PMAxx-treated and untreated samples for each mixture.

**Table S4. Exploratory ROC-derived 2×2 tables for V-PCR outputs versus culture**

To aid interpretability of the continuous V-PCR outputs, we performed an exploratory receiver-operator characteristic (ROC) analysis using culture (positive/negative) as the reference. Youden-index thresholds were derived for two V-PCR measures: (i) absolute viable load (log_10_ PMA+ copies/mL) and (ii) viability percentage. For each threshold, we generated 2×2 contingency tables (V-PCR “viable/non-viable” versus culture) in Table S4A and S4B and reported descriptive performance metrics (sensitivity, specificity, PPV, NPV) in Table S4C. These analyses are presented as descriptive summaries and are not intended to define clinically generalizable decision thresholds.

These cut-offs are presented to aid interpretation of this exploratory clinical-sample application, and not as a clinical validation or finalized decision threshold. Establishing clinical thresholds will require dedicated, prospectively designed studies.

**Table S4A. Absolute viable load (Youden-index threshold = 3.951 log_10_ copies/mL [±8,940 copies/mL]; n = 87)**

|  | Culture positive | Culture negative |
| --- | --- | --- |
| Viable | 37 | 6 |
| Non-viable | 21 | 30 |

**Table S4B. Viability percentage (Youden-index threshold = 0.4269%; n = 87)**

|  | Culture positive | Culture negative |
| --- | --- | --- |
| Viable | 48 | 15 |
| Non-viable | 10 | 21 |

**Table S4C. Performance of viable load and viability percentage testing**

|  | **Sensitivity** | **Specificity** | **Positive Predictive Value** | **Negative Predictive Value** |
| --- | --- | --- | --- | --- |
| Absolute viable load | 0.64 | 0.83 | 0.86 | 0.59 |
| Viability percentage | 0.83 | 0.58 | 0.76 | 0.68 |
